# From rice-husk waste to selective BTEX adsorbents: modified MWCNTs reveal a co-adsorption swing effect and improve field monitoring

**DOI:** 10.1039/d5ra08675b

**Published:** 2026-02-10

**Authors:** Anh H. Q. Le, Hien Y Hoang

**Affiliations:** a Faculty of General Sciences, Ho Chi Minh City University of Natural Resources and Environment Ho Chi Minh City 70000 Vietnam; b Center for Advanced Chemistry, Institute of Research and Development, Duy Tan University 550000 Vietnam hoanghieny@duytan.edu.vn; c Faculty of Environmental and Chemical Engineering, Duy Tan University Danang 550000 Vietnam; d University of Economics Ho Chi Minh City Ho Chi Minh City 70000 Vietnam

## Abstract

In this study, we report for the first time the application of rice husk-derived multi-walled carbon nanotubes (MWCNT) and their three modifications by NaOCl, H_2_SO_4_/HNO_3_, and H_2_O_2_ treatment for the adsorption of gaseous BTEX mixtures (benzene (B), toluene (T), ethylbenzene (E), and xylene (X)). The adsorption capacity followed the order of pristine MWCNT < H_2_SO_4_/HNO_3_-MWCNT < H_2_O_2_-MWCNT < NaOCl-MWCNT, while the preferential uptake of BTEX increased in the sequence B < T < E < X, independent of their initial concentrations, with particularly high selectivity for xylene. Notably, the adsorption efficiency of individual BTEX components was lower than that observed for the mixture. More unexpectedly, stronger adsorbed molecules (*e.g.*, xylene and ethylbenzene), which are typically difficult to desorb under single-gas adsorption, became readily desorbable in the mixture, whereas the opposite trend was observed for weakly bound species such as benzene and toluene. These findings support the DFT-predicted “co-adsorption effect” hypothesis governing inter-species swing adsorption between aromatic hydrocarbons. A field application using NIOSH method 1501 further demonstrated that the developed adsorbents outperformed commercial activated carbon in monitoring BTEX concentrations at a fuel station, especially for xylene. These field observations provide preliminary evidence that substituting rice husk-derived MWCNTs for commercial AC could improve the reliability of the NIOSH method 1501 in determining aromatic hydrocarbons.

## Abstract 2

Rice husk – derived MWCNTs with tailored surface oxidation show selective adsorption of gaseous BTEX, with swing effects enhancing xylene uptake. Field tests (NIOSH 1501) outperform commercial activated carbon.

## Graphical abstract

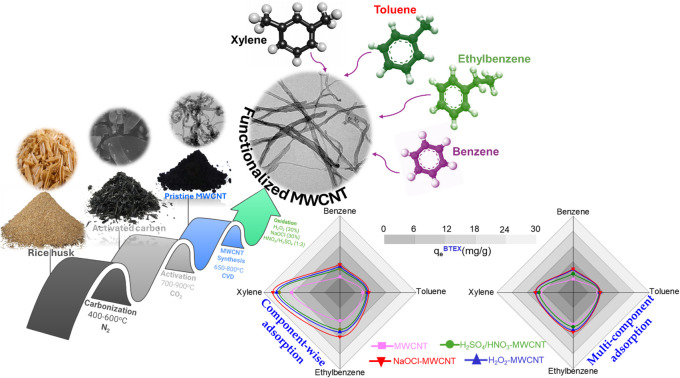

## 1. Introduction

From vehicular emissions to industrial discharge, virtually every individual on Earth is exposed to polluted air on a daily basis. According to the World Health Organization (WHO), approximately 99% of people worldwide are exposed to ambient air that exceeds internationally recognized health-based guidelines. Air pollution has been linked to over 7 million premature deaths annually, making it the second leading risk factor for mortality worldwide, following high blood pressure. In addition to its adverse impacts on human health, air pollution also imposes substantial economic burdens on a global scale. In 2019 alone, air pollution caused economic losses estimated at $8.1 trillion, representing nearly 6.1% of global GDP.^1^ Despite such overwhelming scientific evidence, rapid industrialization and urbanization, along with the expansion of transportation activities, have been hindering all efforts to reduce air pollution globally. According to data provided by Our World in Data, the transportation activities account for approximately 16.2% of total global greenhouse gas emissions, measured in carbon dioxide equivalent (CO_2_e).^2^ Although transportation accounts for a relatively minor fraction of total greenhouse gas emissions, vehicular exhausts contain hazardous compounds that pose significant health risks through acute toxicity and chronic carcinogenic effects upon prolonged exposure. Among these pollutants, particular concern is directed toward volatile organic compounds (VOCs), especially benzene, toluene, ethylbenzene, and xylene (BTEX).^3–5^ Benzene is classified as a Group 1 carcinogen by WHO and the International Agency for Research on Cancer (IARC), indicating sufficient evidence of its carcinogenicity in humans, particularly its association with leukemia and other hematopoietic system disorders. Other compounds of BTEX demonstrate adverse effects on the central nervous system, respiratory tract, hepatic function, and renal systems through both acute and chronic exposure pathways.^6,7^ Compared to common air pollutants such as PM_2.5_, CO_2_, NO_*x*_, SO_2_, or CO, BTEX is more dangerous in terms of cumulative toxicity and the ability to cause cellular damage at the molecular level, even when exposed at low concentrations over a long period of time.^8–10^ Owing to the stability of the π-electron cloud in their aromatic ring structures, BTEX compounds are highly volatile, resistant to degradation, and capable of persisting in the atmosphere for extended periods while dispersing over wide areas. Additionally, a major source of BTEX emissions is the combustion of gasoline in internal combustion engines. These characteristics significantly increase the risk of long-term human exposure, especially in densely populated urban areas with high traffic density, such as in Ho Chi Minh city, Vietnam. Despite the transition to electric and hybrid vehicles being underway, the complete replacement of internal combustion engine vehicles is expected to take time, particularly in developing countries where motorcycles remain the dominant mode of transportation, such as Vietnam. Therefore, the development of technologies capable of managing BTEX continues to be a pressing and high-priority area of research. Although various technologies have been developed to treat BTEX, including catalytic oxidation,^11^ photocatalytic oxidation,^12^ biological oxidation,^13^ adsorption,^14,15^ and plasma destruction,^16^ the use of activated carbon (AC) filters remains the most practical and widely adopted method for controlling pollution caused by this mixture of toxic aromatic hydrocarbons.^17^ Despite the continued use of activated carbon in BTEX removal, advances in nanomaterial science have demonstrated that carbon nanotubes (CNTs), especially when functionalized, possess superior adsorption capacities and selectivity, positioning them as promising next-generation adsorbents.^18^ While CNTs have been extensively studied for the adsorption of BTEX components in aqueous systems,^19^ their potential for adsorbing volatile BTEX compounds in the gas phase remains underexplored. To date, there has been no comprehensive study on the simultaneous gas-phase adsorption of all four BTEX components using CNTs and their functionalized derivatives, nor any comparative assessment of their adsorption efficiencies. This knowledge gap limits the understanding and practical deployment of these carbon nanomaterials in gaseous-phase BTEX treatment, potentially overlooking their promising applicability in air pollution control.

In fact, gas-phase adsorption studies present significantly greater experimental complexity compared to their liquid-phase counterparts. Unlike liquid-phase systems that employ straightforward batch contact methods, gas-phase adsorption experiments necessitate sophisticated instrumentation, including specialized gas collection systems, precision flow control apparatus, and temperature-controlled reaction chambers. The analytical challenges are particularly pronounced, as gas-phase sampling protocols require advanced techniques for accurate quantification of gaseous species concentrations, demanding both specialized analytical equipment and highly trained personnel. Furthermore, the substantial capital investment required for gas-phase adsorption research infrastructure, including vacuum systems, mass spectrometers, and gas chromatography units, creates significant barriers to entry for many research programs. These technical and economic constraints have contributed both to the relative scarcity of gas-phase adsorption studies in the literature and to the limited understanding of gas-phase adsorption mechanisms compared with those in accessible liquid-phase investigations.

In our previous study,^20^ we demonstrated the efficacy of oxygen-functionalized multi-walled carbon nanotubes (MWCNTs) derived from rice husk as an adsorbent for the selective removal of toluene and benzene, and provided mechanistic insights into the adsorption process. However, these adsorption studies were conducted exclusively in the liquid phase. While graphene-based materials, such as CNTs, have been extensively investigated for BTEX adsorption in liquid-phase systems, systematic gas-phase studies—especially those involving multicomponent mixtures and field validation—remain conspicuously scarce. Consequently, direct performance comparison across different adsorption phases is therefore nontrivial, underscoring the need for dedicated gas-phase investigations. Building upon our previous findings on liquid-phase benzene–toluene adsorption^20^ and considering the critical need to address air quality deterioration from transportation-derived BTEX compounds, the present study focuses on evaluating gas-phase adsorption performance for atmospheric BTEX treatment applications. Unlike our previous studies conducted in aqueous environments, this study examines the gas-phase adsorption behavior of all four BTEX components (benzene, toluene, ethylbenzene, and xylene). Notably, in multi-component gas adsorption systems on carbon-based materials, adsorption is generally governed by competitive interactions, whereby different molecules vie for surface adsorption sites, and those with higher affinity or adsorption energy are preferentially adsorbed.^21^ However, conventional competitive adsorption theory cannot fully explain the adsorption–desorption behavior of complex aromatic mixtures such as BTEX, particularly when adsorption preferences differ between mono- and multicomponent systems. Recent density functional theory (DFT)-based simulation has proposed a more complex form of interaction, referred to as “co-adsorption swing behavior”,^22^ wherein species with lower binding energies are initially adsorbed but subsequently displaced by molecules with higher adsorption affinities. This dynamic interplay involves not merely spatial competition, but also fundamental transitions in adsorption states that trigger the desorption of previously bound molecules. Despite these theoretical predictions, to the best of our knowledge, empirical evidence of such behavior in gas-phase systems remains limited, particularly concerning the adsorption of BTEX mixtures on carbon nanotube-based materials. Therefore, the objective of this study extends beyond evaluating the adsorption performance of BTEX mixtures on rice husk-derived MWCNTs, aiming instead to provide experimental validation of this theoretically predicted adsorption behavior.

Furthermore, the present study also evaluates the practical feasibility of implementing the selected CNTs for ambient BTEX capture at a fuel station in Ho Chi Minh city, Vietnam, where elevated vehicular emission concentrations pose significant air quality concerns. One noteworthy point is that the CNTs employed in the present study are derived from rice husk waste, which not only adds value to the adsorbent but also reinforces the environmental relevance of our study by aligning with principles of sustainability and circular economy.

## 2. Experimental section

### 2.1. Adsorbents preparation

**Fig. 1:**
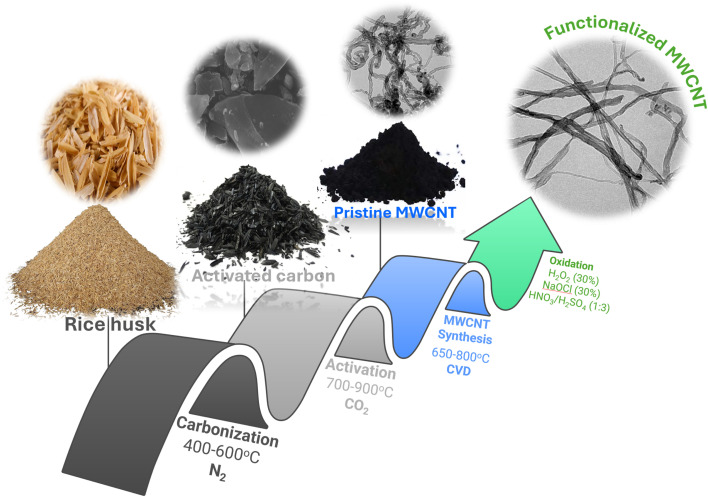
The synthesis pathway of rice husk-derived MWCNTs.

The synthesis of CNT-based adsorbent used in this study involves a multi-step procedure including: (i) conversion of rice husk waste to intermediate AC, (ii) transformation of intermediate AC to MWCNT, and (iii) surface modification of MWCNT using H_2_O_2_, H_2_SO_4_/HNO_3_, and NaOCl as functionalizing agents. The detailed synthetic methodology and characterization protocols have been previously reported in our earlier study,^20^ where the prepared MWCNTs were used for the adsorptive removal of benzene and toluene from aqueous solutions. Briefly, rice husk was first carbonized at 400–600 °C under a continuous nitrogen atmosphere, followed by activation with carbon dioxide at 700–900 °C. Afterward, MWCNTs were synthesized from the obtained AC using the chemical vapor deposition method. The precursor mixture, containing AC, melamine, and iron oxalate, was stirred, vacuum-dried, and subjected to two-step pyrolysis at 650 °C and 800 °C under nitrogen flow. The resulting product was purified with nitric acid and sodium hydroxide to remove residual iron and organics, yielding pristine MWCNTs, which were then oxidized using three different agents: H_2_O_2_ (30%), NaOCl (30%), and HNO_3_/H_2_SO_4_ (1 : 3). Each sample underwent sonication, mild heating, filtration, neutralization, and drying. The resulting functionalized MWCNT — H_2_SO_4_/HNO_3_-MWCNT, H_2_O_2_-MWCNT, and NaOCl-MWCNT were purified and prepared for further adsorption studies. A schematic flowchart of the synthesis of MWCNTs from rice husk is provided in Fig. 1.

### 2.2. Experimental setup

A laboratory-scale system was developed to generate synthetic BTEX gas and evaluate adsorption performance under controlled temperature and flow conditions. Since certified BTEX standard gas is not commercially available, BTEX vapor was generated *in situ via* controlled thermal evaporation of liquid BTEX using purified and dehumidified carrier air, followed by pneumatic transfer into Tedlar gas sampling bags.

**Fig. 2:**
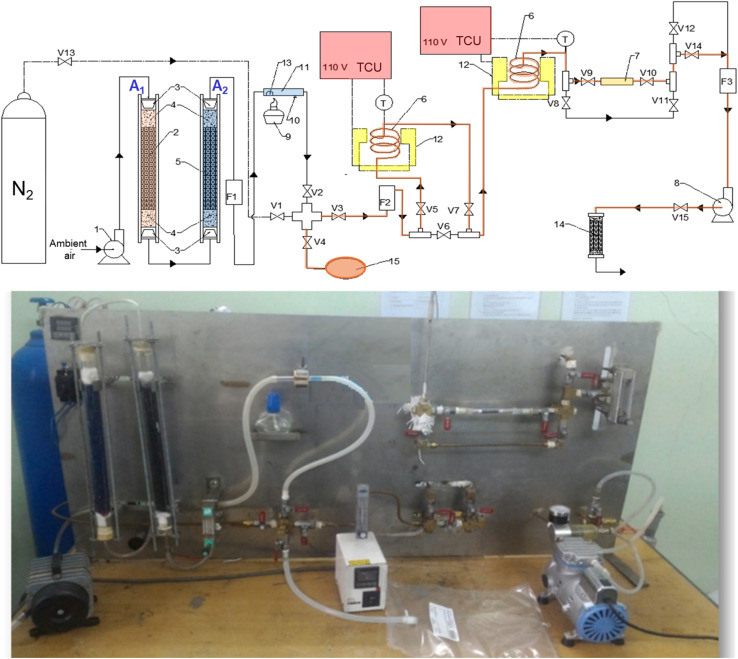
Schematic representation and photograph of the laboratory-scale batch-wise gas adsorption system for BTEX adsorption. Symbols: 1 – delivery pump; 2 – AC-packed column; 3 – rubber stopper; 4 – glass wool plug; 5 – silica gel-packed column; 6 – coiled copper tube; 7 – fixed-bed adsorption column; 8 – vacuum pump; 9 – alcohol lamp; 10 -alignment point; 11 – glass evaporation chamber; 12 – heating chamber with plate heater; 13 – liquid BTEX droplet; 14 – spent adsorption column; 15 – Tedlar gas sampling bag; V1–V15 – brass gas vales; F1–F3 – mass flow controller; TCU – temperature control unit; T – temperature sensor.

The system consists of a temperature-controlled air supply, a gas purification unit, a BTEX vapor generation chamber, a fixed-bed adsorption column, and a pump-assisted gas transport system. Adsorption experiments were conducted using a glass column packed with 50 mg of CNT-based adsorbent. A schematic of the system and detailed specifications of each module are provided in Fig. 2 and SI (Section S1).

#### 2.2.1. Preparation of model BTEX gas for adsorption experiments

Humidity strongly modulates gas adsorption^23–25^ largely through the synergistic effect of water in adsorption. To minimize this interference in BTEX adsorption onto MWCNTs, a dehumidifier was installed at the inlet. Therefore, the air used to generate model BTEX gas was first dehumidified using commercial silica gel and then purified to remove impurities with commercial-flake-form AC. Prior to use, silica gel was thermally dried at 150 °C for 2 hours to eliminate residual moisture. Flaky AC was pretreated by soaking in a 10% HCl solution for 30 minutes at approximately 100 °C, followed by repeated rinsing with distilled water and drying. Both silica gel and AC were stored in a desiccator until use.

A known volume of liquid BTEX was injected into the glass evaporation chamber and vaporized under controlled heating. The generated vapor was pneumatically transferred into a 1.5 L Tedlar gas sampling bag. The target gas-phase concentration was determined from the injected liquid volume and bag volume, and the initial BTEX concentration (C_0_) was quantified by Gas Chromatography-Flame Ionization Detection (GC-FID) immediately prior to adsorption, as summarized in Table S1. Preliminary stability tests using GC-FID indicated that BTEX concentrations in the Tedlar bags remained stable during the adsorption experiments (at least 2 h), with no significant concentration loss observed within the experimental uncertainty. The reproducibility of gas generation was assessed by repeating the vaporization procedure under identical conditions, yielding relative standard deviations below 5% (*n* = 3).

For preparation of BTEX model, initially, valves 1 and 3 are positioned in the closed configuration while valves 2 and 4 are simultaneously opened to establish the primary gas flow pathway. Ambient air is directed through purification silica gel and AC columns, which serve as moisture removal and particulate filtration units, respectively. The purified carrier gas subsequently passes through a glass vaporization chamber (with 100 mm in length and 10 mm in diameter) containing a predetermined quantity of BTEX, where thermal energy is supplied *via* an alcohol burner to achieve controlled vaporization of the target gas. The resulting vapor-laden gas stream is pneumatically transferred into collection bag through the established flow path. Upon achieving the desired gas concentration and volume, all active valves are returned to the closed position to terminate the generation process and isolate the synthetic gas mixture.

#### 2.2.2. BTEX adsorption process

The principle of the adsorption process is that the BTEX gas mixture, after being synthesized and stored in a Tedlar sampling bag, is drawn through the adsorption column by a vacuum pump until the gas in the Tedlar sampling bag is completely depleted. Prior to the adsorption process, the lab-scale system was subjected to a thermal equilibrium stage to achieve a uniform temperature distribution and ensure the establishment of equilibrium conditions throughout the system. During this stage, air was drawn by a vacuum pump (8) through two heating systems (12) maintained at a predetermined temperature. The airflow was regulated and monitored using system-integrated valves and the combined the flow rate, respectively. Remarkably, valves (9, 10) remained closed during the system startup, and the glass tube containing the adsorbent was not yet connected to the system. A preliminary experiment was conducted to monitor variations in temperature and airflow rate during the startup stage, aiming to determine the point at which thermal and flow equilibrium was achieved. The results indicated that after approximately 2 hours of operation, the system reached near-equilibrium conditions, with temperature fluctuations within ±4 °C and flow rate deviations limited to ±2 mL min^−1^.

After the completion of the pre-heating stage, the glass tube adsorption column was connected to the system. Importantly, this tube was preheated in a drying oven set to the same temperature as that used in the adsorption experiments. This procedure was taken to eliminate any temperature differences that could potentially affect the adsorption performance. Afterwards, valves (1, 2, 6, 8, 11, and 12) were closed, while valves (3, 4, 5, 7, 9, 10, 14, and 15) remained open to allow model BTEX gas from the reservoir to be drawn into the adsorption tube by the suction of the vacuum pump (8). The adsorption process was considered complete when the gas sample bag was fully deflated. The adsorption tube was then removed, and spent adsorbent was extracted with CS_2_ solution. The desorption solution was subsequently analyzed by GC-FID to determine the amount of BTEX adsorbed.

The equilibrium adsorption capacity (*q*_e_) was calculated using the following mathematical equation:
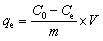
The initial (*C*_0_) and equilibrium (*C*_e_) concentrations of the adsorbate (mg L^−1^) were used to calculate adsorption capacity, where *V* denotes the system volume (L) and *m* the adsorbent mass (g).

The adsorbent selectivity was defined as the adsorption preference ratio between components:
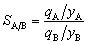
where *S*_A/B_ is the selectivity coefficient of the material for gas A over gas B in the initial BTEX mixture, *q*_A_ and *q*_B_ (mg g^−1^) denote the equilibrium adsorption capacities of components A and B, *y*_A_ and *y*_B_ represent their mole fractions in the initial BTEX mixture.

Following each experimental run, the adsorption system was purged with N_2_ at 120 °C for 30 minutes. Notably, before initiating system operation, N_2_ was also used to perform a leakage test of the system. The system was pressurized to 2 bar, and a soap solution was applied at all joints and connections. The absence of air bubbles over a 30 minute observation period indicated that the system was airtight and suitable for the thermal equilibrium stage. Although pneumatic pumping was used to transport the gas through the adsorption column, the adsorption experiments were conducted under batch-wise conditions using a finite and pre-defined volume of BTEX gas without continuous replenishment.

### 2.3. Desorption experiments

MWCNTs loaded with BTEX mixture were subjected to thermal desorption in a N_2_ flow at temperatures ranging from 260–300 °C. Prior to each desorption run, the adsorption system was purged with N_2_ at 120 °C for 30 min to remove residual gases. The N_2_ flow (50 mL min^−1^) was then directed through the heating zone by opening valves (V1, 3, 5, 7, 8, 11, 13, 14, and 15) and closing valves (V2, 4, 6, 9, 10, and 12), ensuring that the gas reached the target temperature. Once thermal equilibrium was achieved, valves (V8–11) were closed, and valves (V9–10) were opened to allow the preheated N_2_ stream to pass through the spent MWCNTs tube. The desorbed fraction of BTEX was collected in the AC column (14). Following desorption, the desorbed adsorbent was immersed in CS_2_ solution overnight to determine the residual BTEX content. The desorption efficiency was calculated according to the following equation:
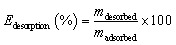
where *E*_desorption_ is desorption efficiency, *m*_desrobed_ donates the BTEX amount desorbed, while *m*_adsorbed_ represents the BTEX amount originally adsorbed.

### 2.4. Field-scale adsorption study of BTEX

Field studies were conducted at the high-traffic urban site in Ho Chi Minh City to evaluate the targeted adsorbent performance for BTEX adsorption under real-world operating conditions. The sampling site was fuel station located at 10°45′40.6″N 106°42′36.5″E. The field-scale study was conducted based on the sampling and determination of BTEX using the NIOSH Method 1501 protocol.

The NIOSH 1501 method is an active air sampling technique for BTEX detection, in which ambient air is drawn through an adsorbent material using a calibrated pump at a controlled flow rate and for a specified duration. Following sample collection, the adsorbed compounds are desorbed using CS_2_ solution, and the target analyte is subsequently quantified using GC-FID.

In this field study, air samples were collected at the fuel station in triplicate. The sampling session lasted 5 hours with an air pump flow rate maintained at 0.1 L min^−1^. Consistent with the lab-scale setup, the field-scale adsorbent was packed into a glass tube, with both ends sealed using glass wool. The tube was connected to the pump's air inlet *via* a silicone pipe. The sampling system was positioned at a height of 1.5 meters above ground level. A parallel control system was also deployed using AC-based adsorbent material under identical conditions. The two systems were placed 1 m apart and operated simultaneously. Meteorological conditions at the gas station during BTEX concentration measurements were documented to complement field observations (as shown in Table S2). All technical specifications of equipment used in BTEX concentration monitoring and field photographs are presented in Table S2 and Fig. S2, respectively.

### 2.5. Characterization and analytical methods

As reported in a previous study, the morphological and physicochemical properties of CNTs-based adsorbent material were comprehensively characterized using various analytical techniques, including X-ray diffraction (XRD), Fourier-transform infrared spectroscopy (FTIR), Field emission scanning electron microscopy (FE-SEM), transmission electron microscopy (TEM), nitrogen adsorption–desorption isotherms, Brunauer–Emmett–Teller (BET) method, DFT-derived pore size distribution, thermogravimetric analysis (TGA) and Raman spectroscopy (HORIBA Jobin Yvon, 632.8 nm excitation, 600 grating, ×50 objective). The BTEX concentrations were determined using GC-FID.

## 3. Results and discussion

### 3.1. Adsorbents characteristics

**Fig. 3:**
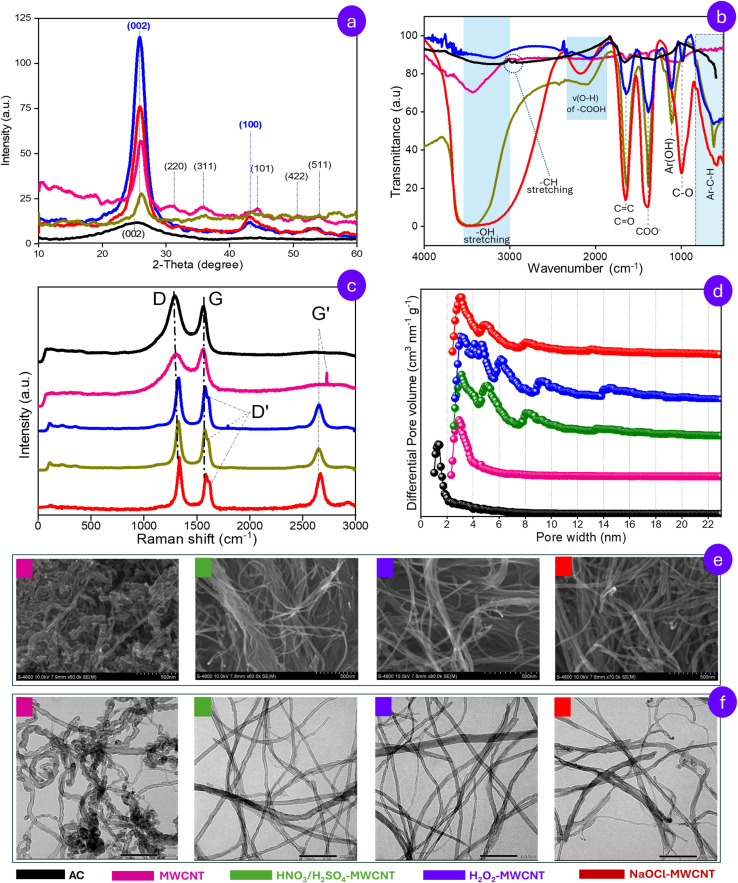
Structural and morphological characterization of rice husk-based carbon adsorbents: (a) XRD patterns; (b) FT-IR spectra; (c) Raman spectra; (d) pore-size distribution curves; (e) FE-SEM images and (f) TEM images.^20^

In our previous study^20^ on adsorptive removal of benzene and toluene from aqueous solutions, the characteristics of rice husk-derived modified MWCNTs were elucidated using XRD, FT-IR, BET, TGA, SEM, TEM, Raman spectroscopy, and DFT-based pore size distribution (PSD) analysis. This study provides a concise overview of these characteristics to offer a more comprehensive understanding of the targeted adsorbent. Specifically, in the crystal phases of rice husk-derived carbons, following CVD treatment, the characteristic absorption peak of AC at 24.38° (002) disappeared, giving way to a new reflection at 26.15° (002) (Fig. 3a), characteristic of carbon-nanotube (JCPDS No. 41-1487). Additional peaks also emerged, including a feature at 44.65° (101), which upon oxidation shifted to = 42.97° (100), consistent with the formation of oxygen-functionalized MWCNTs (JCPDS No. 01-0646). Notably, the intensity of the characteristic diffraction peak at 2*θ* = 26.15° (002) increased markedly after oxidative functionalization, reflecting the unbundling of MWCNT aggregates induced by oxidizing agents. This structural change is evident when comparing the SEM and TEM images of pristine MWCNT with those of its oxidized form (Fig. 3e and f). Remarkably, the oxidation of pristine MWCNTs with the oxidizing agent not only functionalizes the MWCNTs surface but also enhances the purity of the targeted material. Oxidation leads to the removal of residual catalytic impurities—previously evident at characteristic diffraction peaks of Fe-based catalyst such as 31.36° (220), 35.92° (311), 50.68° (422), and 54.43° (511) – yielding modified MWCNTs with significantly improved structural purity.

The functional group analysis (Fig. 3b) reveals that in the 2850–3000 cm^−1^ region, both rice husk-derived AC and pristine MWCNT clearly exhibit characteristic C–H stretching vibrations. Specifically, both materials display a band at approximately 2920 cm^−1^, attributed to the asymmetric stretching of methylene (–CH_2_–) groups. In addition, AC exhibits a peak at 2968 cm^−1^ corresponding to the asymmetric stretching of methyl (–CH_3_) groups, whereas pristine MWCNTs show a peak at around 2850 cm^−1^, characteristic of the symmetric stretching vibration of –CH_2_– (Fig. S1). In contrast, the C–H stretching regions in the spectra of functionalized MWCNT are largely suppressed by a broad absorption band centered around 3500 cm^−1^, which is attributed to strongly interacting hydrogen-bonded hydroxyl groups.^26^ Moreover, a distinct change is observed after oxygen functionalization, where FTIR spectra of oxidized MWCNTs revealed the emergence of a new characteristic peak around 2200 cm^−1^, which can be assigned to the –OH stretch from strongly H-bonded –COOH.^20,27–29^ Additionally, the pronounced enhancement of the absorption band near ∼1658 cm^−1^ in these MWCNTs may be attributed to overlapping contributions from conjugated carbonyl (C=O)^30^ functional groups and the intrinsic C=C stretching vibrations of the graphitic carbon framework.^31^ Especially, the type of oxidant influenced the specific functional groups formed. While phenolic (–OH) groups appeared on MWCNTs treated with H_2_O_2_, alcoholic C–O stretching was observed on those treated with NaOCl. Both of these groups were present on MWCNTs treated with H_2_SO_4_/HNO_3_. Furthermore, the presence of multiple peaks in the 900–500 cm^−1^ range indicates meta-substitution on the MWCNT benzene rings.^32^ The introduction of these oxygen-containing functional groups enhances the surface polarity of MWCNTs and creates multiple adsorption sites for aromatic hydrocarbons. Functionalization simultaneously weakens van der Waals interactions between CNT bundles, improving dispersion. The resulting morphology, characterized by well-separated single filaments, is clearly evident in SEM and TEM images.

The Raman analysis (Fig. 3c) provides further insight into the functionalization degree of MWCNT. Raman spectra of rice husk-based carbon reveal two prominent bands at 1340 cm^−1^ (D-band) and 1581 cm^−1^ (G-band), corresponding to sp^3^ and sp^2^ carbon hybridizations. As opposed to AC, the pristine MWCNT display an additional band at 2720 cm^−1^ (dispersive G′-band) which serves as a spectroscopic signature of the layer number in graphene-like structures.^33^ After modification with NaOCl, H_2_O_2_, H_2_SO_4_/HNO_3_, this band exhibited increased intensity and broadening, consistent with the presence of multiple inner layers. Furthermore, a shoulder peak near 1620 cm^−1^ appeared, corresponding to the D′ band, which indicates structural disorder typical of defective graphite-like materials. At the same time, the D/G intensity ratio (*I*_D_/*I*_G_), derived from baseline-corrected peak intensities to assess the degree of structural disorder relative to graphitic order, exhibited a significant increase across all functionalized MWCNTs, reflecting the conversion of sp^2^ carbon to sp^3^ hybridized carbon and confirming covalent functionalization. Of note, among the oxidizing treatments, NaOCl-functionalized MWCNTs showed the highest *I*_D_/*I*_G_ ratio (1.430), surpassing H_2_O_2_-(1.105) and H_2_SO_4_/HNO_3_-treated samples (1.002), suggesting a greater extent of defect generation under NaOCl oxidation. TGA results indicated that the large structural defects in functionalized MWCNTs markedly reduce their thermal stability, with mass loss exceeding 90% at 700 °C compared to ∼50% for pristine MWCNT. These structural features, along with the abundance of polar functional groups, also explain the higher water retention in the functionalized MWCNTs relative to baseline MWCNT.

Nitrogen adsorption–desorption and pore size distribution analyses further highlight clear differences in structure between these MWCNTs. Although all MWCNTs displayed Type IV isotherms with H3 hysteresis loops, consistent with slit-shaped mesopores formed by platelet aggregates, the pore-size distribution differed markedly. Unmodified MWCNT showed pore widths mainly in the range of 2.3–4.0 nm, whereas oxidation broadened the distribution and introduced additional peaks at ∼4.5, 6.0, and 13 nm in the PSD curves of modified MWCNTS (Fig. 3d). These findings demonstrate that oxidation not only functionalizes the CNT surface but also modifies its pore architecture. However, when considering the specific surface areas individually, the differences among MWCNTs are not statistically significant. Accordingly, BET analysis determined the specific surface area of pristine MWCNTs to be 150.6 m^2^ g^−1^, while oxidation with H_2_SO_4_/HNO_3_, H_2_O_2_, and NaOCl yielded values of 158.0, 179.5, and 187.5 m^2^ g^−1^, respectively. BET surface area and porosity parameters of rice husk-derived carbon materials are summarized in Table S3.

### 3.2. Operation optimization

**Fig. 4:**
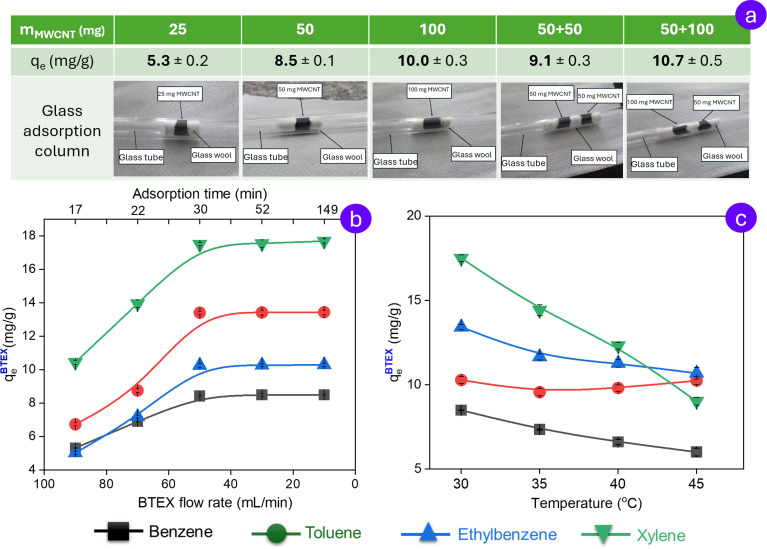
Effect of pristine loading (a), BTEX flow rate and residence time (b), and temperature (c) on the adsorption of individual gases in the BTEX mixture.

To achieve optimal performance conditions, the key operating parameters, including temperature, gas flow rate, and adsorbent loading of the adsorption system, were thoroughly optimized prior to initiating the experimental study. Preliminary investigations of pristine MWCNTs loading effects on benzene adsorption efficiency demonstrated a positive correlation between adsorbent quantity and benzene adsorption capacity. However, beyond a threshold of 50 mg of adsorbent, the improvement in efficiency becomes marginal. Specifically, increasing the pristine MWCNTs mass from 50 mg to 100 mg results in only a 7.1% increase in adsorption efficiency, which was not statistically significant (Fig. 4a). Accordingly, 50 mg of MWCNTs is regarded as the minimum threshold required to ensure effective performance of the adsorption system under investigation. Although multilayer-bed adsorption columns are theoretically expected to enhance adsorption capacity,^34^ preliminary experimental results indicate no notable improvement when comparing single-bed and double-bed configurations. Therefore, a single-bed adsorption column was employed in the investigated system to simplify the experimental setup and minimize operational complexity.

Gas flow rate and contact time exhibit an inverse relationship in the gas adsorption system. Higher gas flow rates reduce contact time, potentially limiting adsorption efficiency due to insufficient interaction between gas molecules and the adsorbent surface. On the contrary, lower former enhances adsorption by increasing the latter, but excessively low rates may compromise process efficiency and feasibility in large-scale applications. This highlights the necessity of contact time optimization for ensuring the effective operation of an experimental gas adsorption system. Empirical evidence indicates that at the gas flow rate lower than 50 mL L^−1^, which corresponds to the contact time maintained below 30 min, the adsorption capacity of pristine MWCNTs demonstrates negligible change across all BTEX components, suggesting equilibrium conditions have been achieved in the adsorption system (Fig. 4b).

In tropical environments with elevated temperatures, such as in Vietnam, it is essential to evaluate adsorption systems under varying thermal conditions to ensure practical applicability. In the system optimization stage, the adsorption efficiency of rice husk-derived MWCNTs for all four investigated gases of BTEX was assessed at four temperature levels ranging from 30 to 45 °C. The results (Fig. 4c) indicated a general decline in adsorption efficiency with increasing temperature, with the most significant reduction observed for xylene. Typically, at elevated temperatures, the kinetic energy of gas molecules increases, which reduces their residence time on the adsorbent surface due to weakened intermolecular interactions. Moreover, higher temperatures shift the adsorption–desorption equilibrium toward desorption, resulting in a decreased amount of gas being adsorbed.

Interestingly, in the case of toluene adsorption, following an initial decline, the adsorption efficiency initially subsequently increases at 40 °C. The rarely encountered phenomenon has been documented in only a limited number of previous studies^35,36^ and could be attributed to activated entry effects. Specifically, the increased kinetic energy at elevated temperature facilitates the diffusion of toluene molecules through the narrow constrictions within the mesoporous network of unmodified MWCNTs, where the abundance of such narrow constrictions is clearly evident in SEM micrographs. This enhanced diffusion allows the molecules to access deeper adsorption sites, thereby increasing the overall adsorption capacity.

Therefore, to ensure optimal performance and high reliability of the adsorption system, the operating conditions should include the BTEX flow rate of 50 mL min^−1^, a single adsorption column packed with more than 50 mg of MWCNTs, and a working temperature of 30 °C.

### 3.3. Component-wise adsorption of BTEX using rice husk-derived modified MWCNTs

**Fig. 5:**
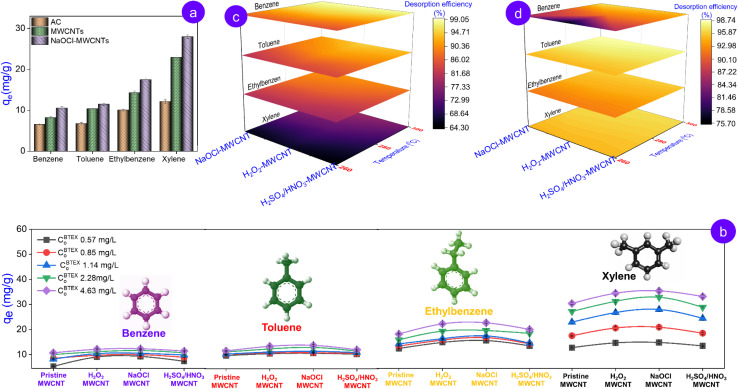
Comparative adsorption performance of BTEX components on rice husk derived adsorbents (a); adsorption efficiency of rice husk-derived MWCNTs as a function of BTEX initial concentrations (b); comparison of desorption efficiency of MWCNTs at varying elevated temperatures under individual- (c) and mixed- (d) BTEX adsorption.

Although rice husk-derived AC demonstrates substantially greater surface area (1039.53 m^2^ g^−1^) relative to MWCNTs (150.66 m^2^ g^−1^) and its functionalized derivatives (an average of approximately 175 m^2^ g^−1^), it exhibits limited BTEX adsorption performance. Notably, the disparity in BTEX adsorption capacity between AC and pristine MWCNTs reaches a maximum in the xylene case, with pristine MWCNTs exhibiting *q*_e_ one point eight times greater than AC (Fig. 5a). The interesting observation can be attributed to the porosity parameter of AC. Accordingly, our previous study revealed that AC possesses a super microporous structure characterized by a pore size of 1.29 nm, creating steric hindrance for BTEX (0.66–0.82 nm in molecular dimensions),^37^ thereby limiting BTEX molecular diffusion into internal pores and reducing overall adsorption capacity despite high surface area values. Conversely, pristine MWCNT and its functionalized derivatives possessing a hollow cylindrical structure, with approximately 6-fold higher pore diameter, exhibited superior BTEX adsorption performance. Additionally, MWCNTs exhibit a superior graphitic surface relative to AC, featuring high uniform π-electron density that facilitates strong π–π interactions with aromatic compounds such as BTEX containing a delocalized π-electron system.

When comparing pristine MWCNT and its oxidized derivatives, the adsorption performance for all four components of BTEX follows the descending order: MWCNT < H_2_SO_4_/HNO_3_-MWCNT < H_2_O_2_-MWCNT < NaOCl-MWCNT, independent of variations in BTEX initial concentration (Fig. 5b). Porosity parameters, such as pore diameter and pore volume, do not govern this trend, as its distribution pattern differs from the sequence of adsorption efficiency. While another parameter, surface area, increases in this order, in our opinion, it provides only a partial explanation for the observed trend.

Taken together, these results indicate that textural properties primarily determine the accessibility of BTEX molecules to the adsorbent surface, acting as a prerequisite for adsorption rather than the dominant controlling factor. Once sufficient pore accessibility is ensured, as in the case of pristine and functionalized MWCNTs, variations in adsorption capacity and selectivity are governed mainly by surface chemistry. In particular, the degree of oxidation and defect density modulate π–π interactions, dispersive forces, and adsorption binding strength, thereby exerting a stronger influence than modest differences in BET surface area.

Although the active site is generally considered an important factor governing adsorption capacity, it is not the primary factor here, as evidenced by H_2_SO_4_/HNO_3_-MWCNT poor efficiency despite a high content of oxygen-functional groups.^20^ This indicates that adsorption is not governed by the quantity of surface functional groups alone, but rather by the nature of surface chemistry and defect structure, which modulate π–π interactions and adsorption binding strength. The relationships between functionalization degree, surface textural properties, and adsorption characteristics of rice husk-derived AC and pristine and functionalized MWCNTs were summarized in Table S3

A consistent trend observed across all tested pristine and functionalized MWCNTs was that the adsorption efficiency of BTEX compounds consistently increased in the order of benzene < toluene < ethylbenzene < xylene irrespective of their concentration (Fig. 5b). The molecular weight and bulkiness adsorbates (ethylbenzene and xylene) are likely contributed to this adsorption order, as larger and bulkier molecules tend to exhibit stronger van der Waals interactions and greater contact area with the adsorbent surface. Moreover, such molecules typically exhibit lower vapor pressures, reducing volatility and enhancing their adsorption onto carbon materials. The molecular weight and vapor pressure order of BTEX are presented in Table S5.

Another reasonable interpretation of the adsorption efficiency order is related to the π-electron density in BTEX molecules, which increases proportionally with the number of alkyl groups attached to the benzene ring. Accordingly, benzene without alkyl substituents has the lowest π-electron density. In contrast, toluene, with a methyl group, exhibits higher electron-rich aromatic ring, while ethylbenzene, containing an ethyl group, shows a slightly greater electron density due to hyperconjugation effects. Finally, xylene, bearing two methyl groups, possesses the highest π-electron density among BTEX compounds, which enhances its π–π interaction potential with MWCNTs.

The high-temperature nitrogen gas desorption experiments provided compelling evidence for the typical adsorption affinity of BTEX compounds on the functionalized MWCNTs. Specifically, as shown by the color mapping (Fig. 5c), although the overall desorption capacity increased with temperature, the established order benzene > toluene > ethylbenzene > xylene remained intact across all modified MWCNTs. Lighter molecules like benzene, with their lower molecular weight and higher vapor pressure, are more easily released. In contrast, increasing the number of alkyl groups on the aromatic ring results in a heavier molecule with enhanced polarity and polarizability. This in turn amplifies the van der Waals and π–π interactions with the CNT surface, effectively trapping the larger molecules within the porous structure. These interactions, further reinforced by electrostatic or π-dipole contributions from surface functional groups of MWCNTs, confine larger aromatic molecules within the pore network, hindering their release even under thermal excitation. This opposing relationship is attributed to the experimentally observed differences in adsorption affinity and retention behavior of BTEX molecules on MWCNTs, rather than to quantitatively determined thermodynamic binding energies, which were not measured in this study.

The study results on component-wise adsorption of BTEX reflect the increasing trend of adsorption affinity, which is directly correlated with molecular weight and the enhanced π–π/van der Waals interactions, a hallmark of the underlying equilibrium-controlled adsorption.

Interestingly, the surface area analysis of MWCNTs following complete desorption indicated a mean increase of approximately 5%. This phenomenon can be attributed to the desorption process at elevated temperature under an inert gas stream. This process effectively served as a thermal cleaning factor, which facilitates the reopening of previously covered micropores, thereby increasing the accessible surface area for N_2_ adsorption during BET measurements (Table S6).^38^ This finding provides an important basis for enabling the recovery of MWCNTs following the BTEX adsorption process.

Although the adsorption experiments were conducted separately for each BTEX compound, the apparent selectivity index of xylene over benzene, toluene, and ethylbenzene remained consistently high, with values occasionally approaching 2.9 (details provided in Table S7). These observations initially suggest that the rice husk-derived MWCNTs exhibit a pronounced affinity and marked preferential adsorption capacity for xylene under the investigated conditions. Clarifying this requires assessing the adsorption capacity of MWCNTs in the BTEX mixture. Moreover, since within the context of environmental and industrial practices, the constituents of BTEX seldom occur in isolation but are usually encountered as coexisting species, the adsorption study should also be conducted on BTEX mixture.

### 3.4. Multi-component adsorption of BTEX using rice husk-derived modified MWCNTs

**Fig. 6:**
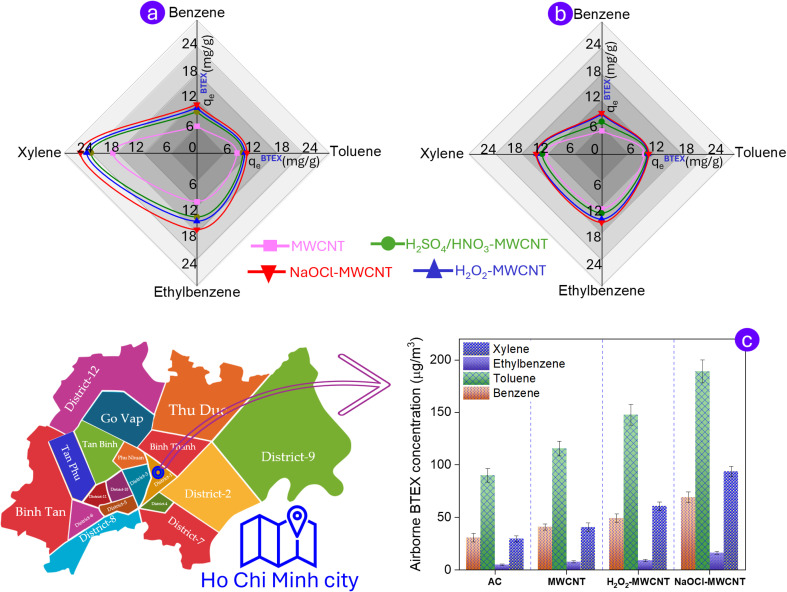
Radar plot illustrating the adsorption efficiency of functionalized MWCNTs under multi- (a) and single-component (b) BTEX adsorption; BTEX concentrations at the surveyed fuel station using different adsorbent materials, together with the relative location of the station (c).

The adsorption results of the BTEX mixture using rice husk-derived modified MWCNTs are comparable to those obtained for component-wise BTEX adsorption. In particular, NaOCl-MWCNT continued to exhibit the highest adsorption efficiency for all four BTEX components, whereas xylene remained the most effectively adsorbed by the modified MWCNTs among all the gases in the BTEX mixture (Fig. 6a). In gas mixtures, small molecules such as benzene, and toluene can easily diffuse into the adsorbent pores. However, they are typically adsorbed less effectively than larger and bulkier molecules substituents such as ethylbenzene and xylene.^39–41^ The observed phenomenon results from a “co-adsorption effect” in the inter-species swing adsorption between aromatic hydrocarbons. Specifically, the adsorbates, characterized by relatively high vapor pressure and small kinetic diameter, such as benzene, tend to diffuse preferentially into the carbon-based adsorbents. Such pre-adsorbed molecules can be classified into three categories based on their adsorption energy: (i) weakly bound molecules that are readily desorbed, (ii) strongly bound molecules requiring a more competitive adsorbate for displacement, and (iii) strongly bound molecules that are difficult to desorb.^22^ Based on the results of density functional theory (DFT)-based modelling approaches, Kumar Vikrant and co-workers concluded that when benzene molecules of the second group are adsorbed first, subsequent gas molecules with higher adsorption energy, such as xylene, can only adsorb over the pre-adsorbed layer of benzene on the graphitic surface of carbon-based adsorbent. This secondary adsorption weakens the interaction between benzene and the graphitized carbon framework, which in turn facilitates the detachment of benzene from the adsorbent. Upon desorption, the desorbed molecules generate the nascent sorption sites with high adsorption enthalpy, which subsequently facilitates the adsorption of later-arriving species such as xylene.^22^ As a result, the later-arriving adsorbates are preferentially retained over the early-arriving adsorbates. This is likely the reason that the amount of xylene adsorbed across all types of MWCNTs was significantly enhanced when co-adsorbed with the gas mixture BTEX (Fig. 6a) compared to when adsorbed individually (Fig. 6b). In contrast, with the other gases in BTEX, the adsorption capacity of the MWCNTs showed no significant change or increased slightly between the two experimental conditions. This finding reveals the clear adsorption selectivity of MWCNTs for gas xylene over the other gases in the multicomponent BTEX system. The selectivity coefficients of the functionalized MWCNTs for xylene over benzene, toluene, and ethylbenzene, on average, were 2.49 ± 0.05, 2.31 ± 0.01, and 1.59 ± 0.04, respectively (details provided in Table S8). The obtained values are 60% greater than those observed in the single-component BTEX system, where xylene showed selectivity over benzene, toluene, and ethylbenzene of only 1.68 ± 0.07, 1.36 ± 0.03, and 0.98 ± 0.01, respectively. This comparison result reaffirmed the synergistic adsorption in facilitating the BTEX removal by rice husk-derived modified MWCNTs.

To gain further insight into BTEX adsorption with rice husk-derived modified MWCNTs, we also investigated the desorption behavior of MWCNTs following the multi-component adsorption of the BTEX mixture and compared the results with those obtained from the single-component adsorption of BTEX. As shown by the color mapping in Fig. 5c and d, the desorption behavior of MWCNTs in the case of multi-component adsorption differs markedly from the case of single-component one. Benzene exhibits the lowest and most strongly reduced desorption efficiency, whereas the other gases in the BTEX mixture display improved desorption. Interestingly, xylene shows the greatest enhancement, reaching a level comparable to the remaining gases.

Evidently, in the context of component-wise adsorption, the desorption is directly governed by the adsorption binding energy between the adsorbent surface and the adsorbate molecules, and, consequently, directly reflects the desorption order of benzene > toluene > ethylbenzene > xylene. Since each molecule follows its own desorption pathway, nearly all adsorbed gases can be released when appropriate conditions of temperature and vacuum are provided. In contrast, for multi-component adsorption, the desorption is also influenced by the adsorption mode. Here, the benzene retained in the adsorbate following BTEX mixture adsorption is dominated by the third group (as mentioned above) with strong binding and low desorbability. Meanwhile, other gas species such as xylene adsorb onto the pre-adsorbed benzene layer *via* weak interactions, enabling their more facile release than that in a single-component system. This observation not only provides a comprehensive picture of the adsorption of BTEX mixture by rice husk-derived MWCNTs but also further supports the theory about inter-species swing adsorption between BTEX proposed by Kumar V. and co-workers in their previous study through DFT analysis.

Overall, the markedly different desorption behaviors between single- and multi-component systems provide direct experimental evidence for inter-species competition and layered adsorption, thereby reinforcing the proposed inter-species swing adsorption mechanism.

Following the confirmation of the CNTs adsorption efficiency for BTEX under controlled laboratory conditions, it was essential to assess their performance in real-world settings. To this end, a field-scale adsorption study of BTEX using the targeted adsorbents was conducted to evaluate the operational stability, adaptability to variable environmental conditions, and the overall feasibility of deploying the targeted material in practical applications.

### 3.5. On-site measurement of BTEX content at a fueling station

Ensuring practical applicability is central to the development of environmental treatment materials. Field measurements are thus required to validate their performance and to evaluate adsorption efficiency under realistic conditions, where BTEX occurs at low concentrations and is influenced by temperature, humidity, and coexisting impurities. For *in situ* gas sampling, passive samplers are often used for long-term monitoring, but their reliance on uncontrolled diffusion makes it difficult to regulate gas flow through the adsorbent, leading to large uncertainties and limiting comparisons of material performance. To overcome these limitations, this study employed an active sampling approach (NIOSH method 1501), in which ambient air was pumped through sorbent tubes containing rice husk-derived modified MWCNTs. This approach, analogous to our laboratory protocol, enables precise control of flow rate and sampling duration, thereby facilitating a direct evaluation of adsorption capacity relative to commercial adsorbents. For the field study, a public petrol station (its visual location is depicted in Fig. 6c) was selected as the sampling site, representing a typical source of BTEX emissions from gasoline evaporation and fueling activities. The site provides high and strongly fluctuating BTEX concentrations over the course of a day, offering a realistic setting to assess the performance of MWCNTs under complex environmental conditions.

At petrol stations, toluene and benzene are typically the most abundant BTEX components, often present at higher concentrations than the others. While most monitoring studies report benzene levels exceeding those of toluene, our results—and those of several other studies^42–45^—show the opposite trend, with benzene concentrations lower than toluene and even xylene, as illustrated in Fig. 6c. This raises the question of whether the presence of strongly adsorbing species such as xylene could induce competitive displacement, potentially leading to reduced retention or underestimation of benzene. It is noteworthy that the lower benzene concentrations relative to toluene and xylene observed in the field measurements are not unique to MWCNT-based adsorbents. A similar trend is also evident when AC, a conventional sorbent widely used in active BTEX sampling methods such as NIOSH Method 1501, was employed (Fig. 6c). This observation indicates that the reduced benzene levels detected during sampling cannot be solely attributed to competitive displacement by xylene on MWCNTs. Instead, this discrepancy is primarily attributable to the implementation of vapor recovery systems, which markedly reduce benzene evaporation during refueling.^45^ Fuel composition and additives also play a role, as certain petrol blends and solvents contain higher proportions of toluene. Furthermore, climatic factors and monitoring conditions contribute to the variability,^43^ since the differing vapor pressures and volatilization properties of benzene and toluene cause their ambient concentrations to vary with temperature, wind direction, and station ventilation.

In parallel, the field experiments further substantiate the potential of rice husk-derived MWCNT-based adsorbents for BTEX removal, with adsorption capacities following the order commercial AC < pristine MWCNT < H_2_O_2_-MWCNT < NaOCl-MWCNT. When the sorbent tubes containing NaOCl-MWCNT were employed, the concentrations of benzene, toluene, ethylbenzene, and xylene at the fuel station were found to be 69.1 ± 5.2 µg m^−3^, 189 ± 11 µg m^−3^, 16.3 ± 1.5 µg m^−3^, and 93.6 ± 5.1 µg m^−3^. Accordingly, the toluene concentration found in this study (∼189 µg m^−3^) falls within the broad range reported for petrol stations in international surveys, depending on station activity. Similarly, the benzene concentration (∼69 µg m^−3^) lies within previously reported ranges of several tens to a few hundred µg m^−3^, though higher levels have often been documented at sites where fuels or operational conditions involve elevated benzene content. At the same time, ethylbenzene (∼16 µg m^−3^) represented the lowest fraction of the BTEX mixture, a pattern consistent with the compositional profiles frequently reported at petrol stations. In contrast, the xylene concentration exhibited an unexpected deviation from previously reported patterns. Several factors may underlie this anomaly, including fuel composition, operational conditions at the station, and what may be a selective interaction of xylene with carbon-based adsorbents used. Notably, when commercial AC was used as the adsorbent following the NIOSH method 1501, xylene was almost entirely depleted, representing the lowest fraction among the four measured gases of BTEX. However, replacement of AC with NaOCl-MWCNT resulted in a marked increase in xylene by more than three times, which is consistent with the strong intrinsic selectivity of MWCNTs toward xylene, as independently demonstrated in controlled laboratory adsorption experiments. This enhancement, therefore, reflects a genuine material-dependent adsorption preference rather than an artifact arising from benzene intrusion or loss. Nevertheless, under multicomponent field conditions, competitive interactions among BTEX species may still contribute to the overall adsorption behavior to a limited extent.

Although humidity was minimized in laboratory experiments using silica gel to elucidate the intrinsic mechanism and adsorption efficiency of BTEX on MWCNT-based adsorbents, it should be acknowledged that real atmospheric conditions inevitably contain water vapor, with field relative humidity ranging from 55.7 to 59.2%, as described in Table S3. The MWCNTs employed in this study were oxygen-functionalized, introducing polar surface groups, while extensive graphitic domains were preserved and remained the dominant adsorption sites for aromatic hydrocarbons *via* π–π stacking and van der Waals interactions. Under humid conditions, water vapor is expected to preferentially interact with oxygen-containing functional groups, whereas BTEX molecules primarily adsorb on graphitic surfaces. This results in site-specific adsorption rather than direct competition for identical sites. Importantly, all investigated adsorbents were exposed to identical ambient humidity during field measurements; therefore, the observed differences in adsorption performance reflect intrinsic material selectivity rather than humidity-induced artifacts. The consistent xylene-preferential adsorption observed in both laboratory and field experiments further supports the validity of the proposed adsorption mechanism under realistic atmospheric conditions. Nevertheless, a systematic investigation of the effects of relative humidity on BTEX adsorption kinetics and equilibrium is warranted and will be the focus of future work.

It should be noted that the commercial AC and the investigated MWCNTs are not texturally equivalent materials. One material is mainly microporous with a high surface area, while the other possesses a hollow porous structure with larger effective pore diameters and a lower surface area. Therefore, the comparison highlights practical adsorption performance under realistic conditions rather than intrinsic adsorption capacity normalized by surface area.

Overall, these field observations provide preliminary evidence that substituting rice husk-derived MWCNTs for commercial AC could improve the reliability of the NIOSH method 1501 in determining aromatic hydrocarbons.

## 4. Conclusion

Beyond their effective removal of benzene and toluene in solution, rice husk-derived modified MWCNTs also exhibit strong adsorption capacity for BTEX gas mixture, with pronounced selectivity toward xylene. For all four constituent gases, the adsorptive removal efficiency decreases in the order pristine MWCNT < H_2_SO_4_/HNO_3_-modified MWCNT < H_2_O_2_-modified MWCNT < NaOCl-modified MWCNT. Notably, the adsorption capacity of these materials declines when moving from single- to multi-component adsorption of BTEX, supporting the previously proposed “co-adsorption effect” hypothesis about inter-species swing adsorption between aromatic hydrocarbons. In which weaker adsorbed molecules (benzene) are replaced by stronger ones (xylene), accompanied by enhanced adsorption of the latter. In addition, the contrasting desorption behavior of the spent adsorbents between single- and mixed-gas adsorption further reinforces this hypothesis. Moreover, results from field experiments confirm that these adsorbents effectively remove BTEX under practical conditions. The consistency between field and laboratory findings underscores that rice husk-derived modified MWCNTs hold considerable promise for controlling and mitigating BTEX pollution in urban air. Future studies will extend this work by investigating the adsorption dynamics and thermodynamic aspects of BTEX on modified MWCNTs under continuous-flow conditions, providing deeper insight into rate-limiting steps and scale-up potential for air pollution control.

## Author contributions

Anh H. Q. Le: project administration, experiments, formal analysis, reviewing & editing, data curation. Hien Y Hoang: supervision, writing – original draft, writing – review & editing, software, methodology, validation.

## Conflicts of interest

The authors declare that they have no known competing financial interests or personal relationships that could have appeared to influence the work reported in this paper.

## Supplementary Material

RA-016-D5RA08675B-s001

## Data Availability

The authors confirm that the data supporting the study findings are available within the manuscript. Raw data that support these findings are available from the corresponding author, upon request. Supplementary information (SI) is available. See DOI: https://doi.org/10.1039/d5ra08675b.
