# Supplementary material for: From rice-husk waste to selective BTEX adsorbents: modified MWCNTs reveal a co-adsorption swing effect and improve field monitoring
Source: RSC Adv. 2026 Feb 10;16(9):8105–18. doi: 10.1039/d5ra08675b (PMC12887758; doi:10.1039/d5ra08675b)
Supplement: RA-016-D5RA08675B-s001 [file RA-016-D5RA08675B-s001.pdf]

Supplementary Information (SI) for RSC Advances.
This journal is © The Royal Society of Chemistry 2026

SUPPLEMENTARY DATA

From rice-husk waste to selective BTEX adsorbents: modified MWCNTs reveal a co-adsorption swing effect and improve field monitoring

Anh H.Q. Le[a], *Hien Y Hoang*[b,c]

[a] Faculty of General Sciences, Ho Chi Minh city University of Natural Resources and Environment, Ho Chi Minh city 70000, Vietnam

[b] Center for Advanced Chemistry, Institute of Research and Development, Duy Tan University, 550000, Viet Nam

[c] Faculty of Environmental and Chemical Engineering, Duy Tan University, Danang, 550000, Viet Nam

Section S1: Experimental setup

A laboratory-scale treatment system was designed to investigate the effects of temperature, gas flow rate, and other operational parameters on the adsorption efficiency of BTEX. Since standard sample gas containing BTEX is not commercially available, it must be generated in situ. The synthetic gas generation protocol involves the controlled vaporization of BTEX solution using heat and purified carrier air, followed by pneumatic transfer of the resulting vapor into storage bags via pump-assisted flow. The air used in this process is first dehumidified and filtered to ensure purity.

To accommodate these specialized requirements, the experimental apparatus comprises seven integrated subsystems: temperature control module, valve regulation system with flow metering capabilities, air purification unit, synthetic gas generation chamber, BTEX adsorption reactor, post-treatment gas collection assembly, and pneumatic pumping system for gas transport. The complete system configuration and process flow are illustrated schematically in Figure 2 of the manuscript.

The temperature control module consists of a plate heater, an inner spiral tube for air conduction, a pair of temperature sensors, and a central controller. Air flows through the spiral tube and is heated externally by the plate heater. The controller receives signals from a temperature sensor located at the module outlet and adjusts the heating power to maintain the preset temperature. The outer shell of the module is made of stainless steel, the space between the shell and the heating plate is filled with glass wool for insulation. The air pipe used in the lab-scale system was made of copper with an internal diameter of 5mm. The heating system is positioned downstream of the adsorption system support frame.

The air purification unit comprises two packed glass columns (each 500 mm in length and 50 mm in diameter, with both ends sealed using fiberglass), designated as column A_1 and column A_2, loaded with silica gel and flaky AC, respectively. The packed length of each column was approximately 40 cm. Silica gel is substituted upon saturation, as indicated by a color change to pink. Flake-activated carbon, which is non-reusable under the current system configuration, is routinely replaced every 10 days. The spent silica gel is regenerated via thermal desorption at 110 °C for 24 hours, whereas the activated carbon is discarded without regeneration.

The synthetic gas generation chamber consists of a glass tube (length: 100mm, internal diameter: 10mm) designed for controlled dispensing of BTEX solution. The tube is connected to a Tedlar gas sampling bag via a four-way valve and heated using an alcohol burner to facilitate BTEX evaporation. The Tedlar gas sampling bag also serves as a post-treatment gas collection assembly.

The BTEX adsorption reactor is a glass tube 100mm in length and 7 mm in inner diameter, filled with 50 mg of CNT-based materials. The adsorbent is secured in place by glass wool, forming

an effective adsorption column of approximately 5–6 mm in length. Each unit is pre-prepared, evacuated, and sealed at both ends for storage and preservation.

Two types of pumps are employed in a pneumatic pumping system: a delivery pump and a vacuum pump. The delivery pump is responsible for generating vapor from the BTEX solution, while the vacuum pump creates the necessary pressure differential to draw BTEX vapor from the gas sampling bag through the adsorption column.

Technical specifications and detailed illustrations of the equipment used in the lab-scale system for adsorbing BTEX using MWCNT are provided in Table S2.

Table S1 – Injected BTEX volume and calculated initial BTEX concentration in the gas sampling bag

**Injected BTEX volume (μL)**	**Gas bag volume (mL)**	**Initial BTEX concentration, C_0 (mg L^{-1})**
1.0	1500	0.53
1.5	1500	0.92
2.0	1500	1.11
4.0	1500	2.25
8.0	1500	4.49

Table S2 - Technical specifications and detailed illustrations of the equipment used in the lab- and field - scale system for adsorbing BTEX using rice husk–based carbons

**For lab-scale system**	
Instrument	Technical specification
Temperature control unit	Brand: Fine<br>Model: PHP-101<br>Accuracy: 0.02 – 0.5 °C<br>Power supply: 100 V / 50–60 Hz<br>Temperature range: 0 – 200 °C
Vacuum pump	Brand: Rocker; Model: 300<br>Type: Oil-free vacuum pump<br>Operation: Quiet and stable<br>Pumping speed: 18 L/min<br>Ultimate vacuum: up to 670 mmHg<br>Vacuum gauge: Integrated<br>Accessories: Built-in moisture and dust trap<br>Power supply: 220 V / 50 Hz
Delivery pump	Brand: Resun<br>Model: ACO-004<br>Power supply: 220 V / 50 Hz<br>Power consumption: 58 W<br>Air flow rate: 75 L/min<br>Pressure: 0.028 MPa
Mass flow controller (F1)	Brand: Kofloc

	Model: RK1710<br>Target Fluid: Air. Nitrogen（N2）<br>Effective Scale: 10: 1<br>Connector Size: Rc1/8<br>Pressure Resistance Guarantee: 0.5MPa<br>Heat Resistant Temperature: 60°C
AC- and silica gel-packed columns	Column type: Packed glass column<br>Dimensions: 500 mm (length) × 50 mm (diameter)<br>Packed length: ~400 mm<br>End sealing: Fiberglass plugs
Tedlar gas sampling bag	Brand: Merck<br>Capacity: 1.6L **L × W**<br>L × W: 9 in.× 9 in.
**For field-scale system**	
Sampling pump	Brand: Hario<br>Model: HSP-500. Code 400-00500<br>Flow rate setting range (L/min): 0.050 - 0.500<br>Instant flow rate setting range (L/min): 0.010 - 0.750<br>Pressure resistance: 12kPa or more<br>Flow rate accuracy: 5% compared to set flow rate value<br>Integrated flow rate setting range: 0 - 1999.99L
Weather Meter	Brand: Kestrel<br>Model: 5500
Multi-Function Ventilation Meter	Brand: VelociCalc<br>Model: 9565-A

Table S3 - Meteorological conditions recorded at the gas station during sampling for BTEX concentration measurements

Sampling period	Phase I	Phase II	Phase III
**Sky conditions**	Cloudy at night (no precipitation); partly cloudy/sunny during the day; light winds	Cloudy night (no rainfall); partly cloudy and sunny day; light winds	Cloudy at night (no precipitation); partly cloudy/sunny during the day; light winds
**Temperature (ºC)**	32.6 –33.7	30.0 – 34.5	33.6 – 34.6
**Relative humidity (%)**	55.7 –59.2	60.3 – 62.9	58.2 – 61.4
**Atmospheric pressure**	1002.4 –1004.5	1009.1 – 1010.1	1003.6 – 1005.7

Table S4 – Correlation between structural disorder (I_D/I_G), surface textural properties, and adsorption-related characteristics of pristine and functionalized MWCNTs and activated carbon.

Adsorbent	I_D/I_G	Porosity	Surface area (m_2/g)	Total pore volume (cm^3/g)	Average pore diameter (nm)	BTEX adsorption capacity (mg/g)			
						**B**	**T**	**E**	**X**
**AC**	1.32	Supermicroporous (1-2 nm)	1039.53	0.97	1.29	6.53	6.74	10.04	12.14
**Pristine MWCNT**	0.85	Mesoporous (2.3 -4.0 nm)	150.66	0.41	6.06	8.18	10.34	14.27	22.95
**H_2SO_4/HNO_3 -**	1.002	Mesoporous	158.01	0.36	6.12	9.85	10.94	14.58	24.46

**MWCNT**		(2.3-13.0 nm)							
**H_2O_2 - MWCNT**	1.105	Mesoporous (2.3-13.0 nm)	179.47	0.34	7.20	10.37	11.15	16.35	26.75
**NaOCl - MWCNT**	1.430	Mesoporous (2.3-13.0 nm)	187.50	0.31	6.62	10.53	11.45	17.45	27.99

Table S5 - Physicochemical properties of BTEX compounds

**Characteristics**	**Benzene**	**Toluene**	**Ethylbenzene**	**Xylene**
**Formula**	C_6H_6	$C_6H_5CH_3$	$C_6H_5CH_2CH_3$	$C_6H_4(CH_3)_2$
**Polarity**	Non-polar	Non-polar	Non-polar	Non-polar
**Molecular weight (g/mol)**	78.12	92.15	106.18	106.18
**Density (g/ml)**	0.876	0.867	0.867	0.868
**Vapor pressure at 25°C (mmHg)**	95	30	10	7–8
**Boiling point (°C)**	80	111	136	137–144

Table S6 - Average volume of specific surface of rice husk–derived MWCNTs prior to BTEX adsorption and following N_2 desorption

Adsorbents	Average volume of specific surface (m^2/g)	
	Prior to adsorption	Following desorption
**H_2SO_4/HNO_3 - MWCNT**	158.01	165.79
**H_2O_2 - MWCNT**	179.47	185.23
**NaOCl -MWCNT**	187.50	193.86

Table S7 - Apparent selectivity indices of rice husk-derived MWCNTs toward BTEX in their component-wise adsorption.

**Adsorbent type**	**Initial concentration of BTEX (mg/l)**	**Selectivity X/B**	**Selectivity X/T**	**Selectivity X/E**
Pristine MWCNT	*0.57*	2.42	1.32	1.03
	0.85	2.06	1.70	1.30
	1.14	2.81	2.22	1.61
	2.28	2.71	2.43	1.71
	4.63	2.84	2.62	1.67
H_2SO_4/HN_3 -MWCNT	*0.57*	1.83	1.31	1.00
	0.85	2.08	1.75	1.30
	1.14	2.48	2.24	1.68
	2.28	2.70	2.51	1.56
	4.63	2.91	2.76	1.66
H_2O_2	*0.57*	1.63	1.40	0.99
	0.85	2.12	1.91	1.30

-MW[illegible]	*1.14*	2.58	2.40	1.64
	2.28	2.81	2.52	1.61
	4.63	2.85	2.57	1.55
NaOCl - MWCNT	*0.57*	1.60	1.39	0.95
	0.85	2.10	1.92	1.25
	1.14	2.66	2.45	1.60
	2.28	2.83	2.53	1.68
	4.63	2.88	2.57	1.56

Table S8 - Selectivity indices of rice husk-derived MWCNTs toward BTEX in their multi-component adsorption.

**Adsorbent type**	**Initial concentration of BTEX (mg/l)**	**Selectivity X/B**	**Selectivity X/T**	**Selectivity X/E**
**Pristine MWCNT**	0.57	3.15	2.11	1.73
**H_2SO_4/HNO_3 - MWCNT**		2.58	2.30	1.65
**H_2O_2 - MWCNT**		2.46	2.30	1.61
**NaOCl -MWCNT**		2.42	2.32	1.52

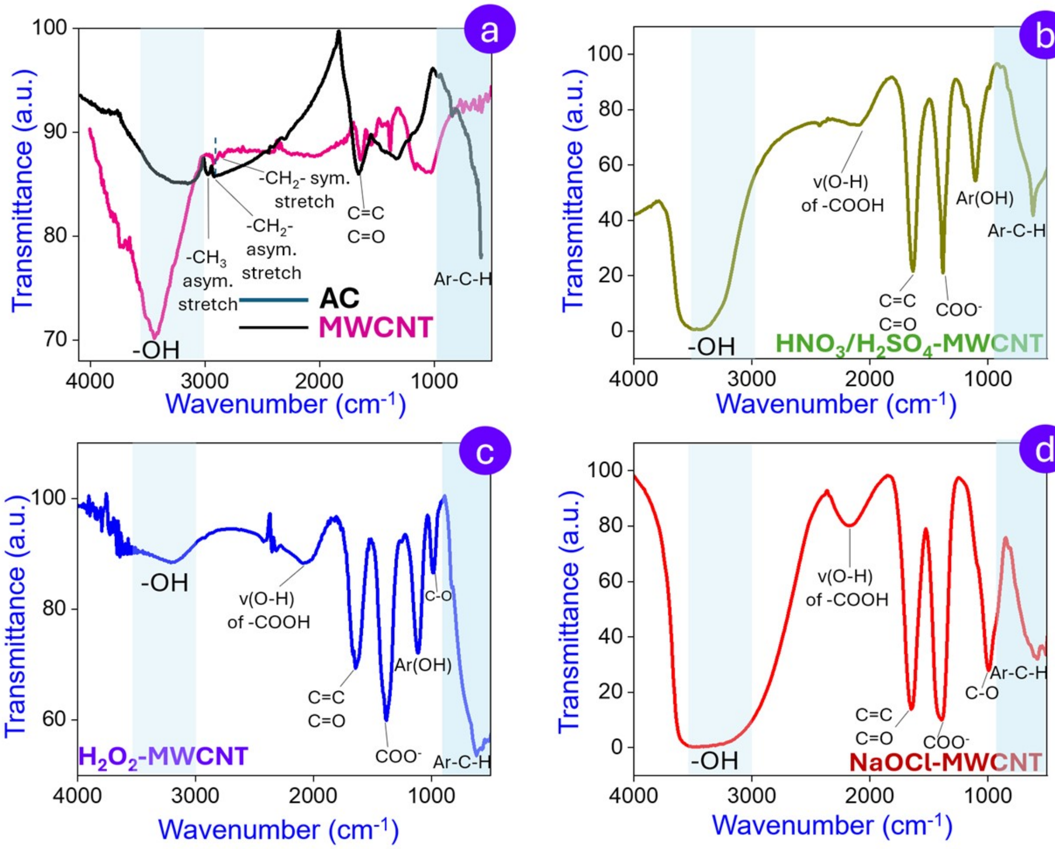

Figure S1- FT-IR spectra of rice husk-derived AC and pristine MWCNTs (a), and HNO_3/H_2SO_4- (b), H_2O_2- (c), and NaOCl (d) functionalized MWCNTs

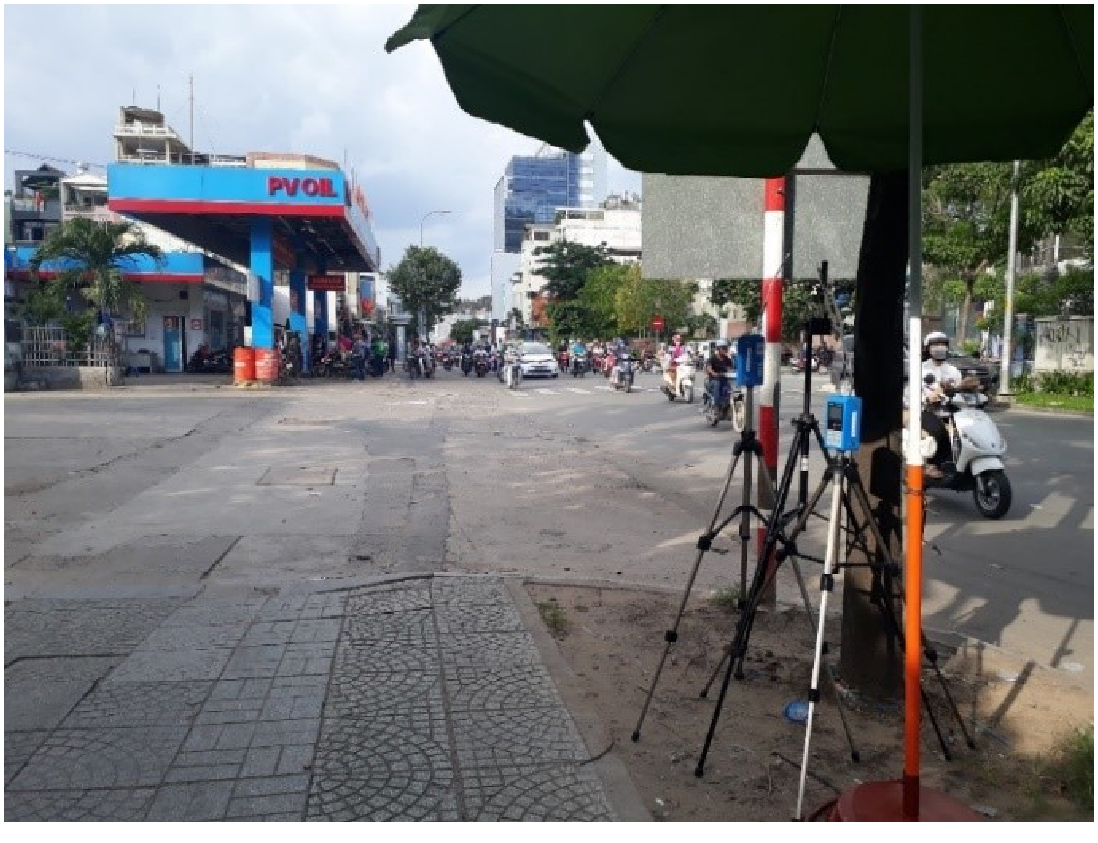

Figure S2 - Field photograph of BTEX concentration monitoring conducted at the fuel station
